# Spred2-deficiecy Protects Mice from Polymicrobial Septic Peritonitis by Enhancing Inflammation and Bacterial Clearance

**DOI:** 10.1038/s41598-017-13204-7

**Published:** 2017-10-09

**Authors:** Junya Itakura, Miwa Sato, Toshihiro Ito, Megumi Mino, Soichiro Fushimi, Sakuma Takahashi, Teizo Yoshimura, Akihiro Matsukawa

**Affiliations:** 1 Department of Pathology and Experimental Medicine, Graduate School of Medicine, Dentistry and Pharmaceutical Sciences, Okayama University, Okayama, 700-8558 Japan; 2 Department of Immunology, Nara Medical University, Nara, 634-8521 Japan

## Abstract

Sepsis is an infection-induced systemic inflammatory syndrome and a major cause of death for critically ill patients. Here, we examined whether the absence of Sprouty-related EVH1-domain-containing protein 2 (Spred2), a negative regulator of the Ras/Raf/ERK/MAPK pathway, influences host defense against polymicrobial sepsis (PMS) induced by cecal ligation and puncture (CLP). Compared to wild-type mice, Spred2^−/−^ mice exhibited higher survival rates with increased level of leukocyte infiltration and local chemokine production and reduced plasma and peritoneal bacterial loads after CLP. The MEK inhibitor U0126 significantly reduced LPS-induced chemokine production by Spred2^−/−^ resident macrophages *in vitro*, and decreased CLP-induced leukocyte infiltration *in vivo*. Spred2^−/−^ resident macrophages, but not neutrophils or elicited macrophages, exhibited increased phagocytic activity. Interestingly, surface expression of complement receptor 1/2 (CR1/2) was increased in Spred2^−/−^ resident macrophages in response to lipopolysaccharide in a manner dependent on the ERK/MAPK pathway, and blocking CR1/2 *in vivo* resulted in reduced leukocyte infiltration and increased bacterial loads after CLP. Taken together, our results indicate that Spred2-deficiency protects mice from PMS via increased activation of the ERK/MAPK pathway and subsequent increase in innate immune responses. Thus, inhibiting Spred2 may present a novel means to prevent the development of PMS.

## Introduction

Severe sepsis is still a major cause of death for critically ill patients, with an estimated incidence of nearly 1,000,000 cases and 200,000 deaths annually in the United States alone^1^. Despite advances in supportive care, the mortality rate of sepsis (30–50%) remains unchanged for the past three decades and is the leading cause of death in noncoronary intensive care unit^2^. Sepsis is a huge burden for healthcare systems, and the management and treatment of sepsis remain challenging.

The pathophysiology of sepsis involves dysregulation of the inflammatory response, coagulation abnormalities and multiple organ failure, resulting from the actions of numerous mediators belonging to the complement system, coagulation cascade, fibrinolysis cascade and cytokines/chemokines^1,3^. Following pathogen infection or tissue damage, the innate immune system recognizes microbial components by pattern recognition receptors (PRRs), and induces host responses through the activation of intracellular signal transduction pathways^4^.

There is a great interest in studying the cellular processes and signaling pathways during sepsis. We have thus far demonstrated using a murine model of septic peritonitis that signal transducer and activator of transcription (STAT) and its negative regulator suppressors of cytokine signaling (SOCS) have a significant impact on innate immune responses during sepsis^5–8^. During sepsis, bacterial components that include lipopolysaccharide (LPS) activate several intracellular signaling pathways, including the NF-κB and MAPK pathways^9,10^. The MAPK family is composed of the c-Jun N-terminal kinase (JNK)-1/2, p38 and extracellular signal-regulated kinase (ERK)-1/2^11^. Among them, activation of ERK was detected in LPS-induced endotoxemia and septic peritonitis induced by cecal ligation and puncture (CLP) which closely resembles the clinical course of polymicrobial peritonitis, bacteremia, and systemic sepsis in humans^9,12–14^. Thus, the ERK pathway appears to play an essential role in the pathogenesis of sepsis and septic shock.

Sprouty-related EVH1-domain-containing proteins (Spreds) are a family of proteins that inhibit Ras-dependent ERK signaling^15^. As the ERK/MAPK pathway is activated in sepsis and septic shock, endogenous Spred proteins may be involved in the regulation of immune responses during sepsis. However, the physiological functions of Spred proteins in sepsis remain to be elucidated. Spred1 and 3 are selectively expressed in the brain and cerebellum, whereas Spred2 is ubiquitously expressed in various tissues, including the colon, lung and spleen^16,17^. We recently demonstrated that Spred2-deficiency exacerbated LPS-induced lung inflammation with increased leukocyte infiltration by up-regulating the ERK/MAPK pathway^18^, leading us to hypothesize that interfering Spred2 may be useful to protect hosts against microbial infection. In the present study, we demonstrate, for the first time, that Spred2-deficiency protects mice from CLP-induced polymicrobial sepsis (PMS). Interestingly, phagocytic activity was significant enhanced in Spred2-deficient macrophages, via augmented cell-surface expression of complement receptor 1 and 2 (CR1/2). Thus, Spred2 may represent a novel therapeutic target to prevent the development of PMS.

## Results

### Enhanced innate immune responses in Spred2^−/−^ mice after CLP

**Figure 1:**
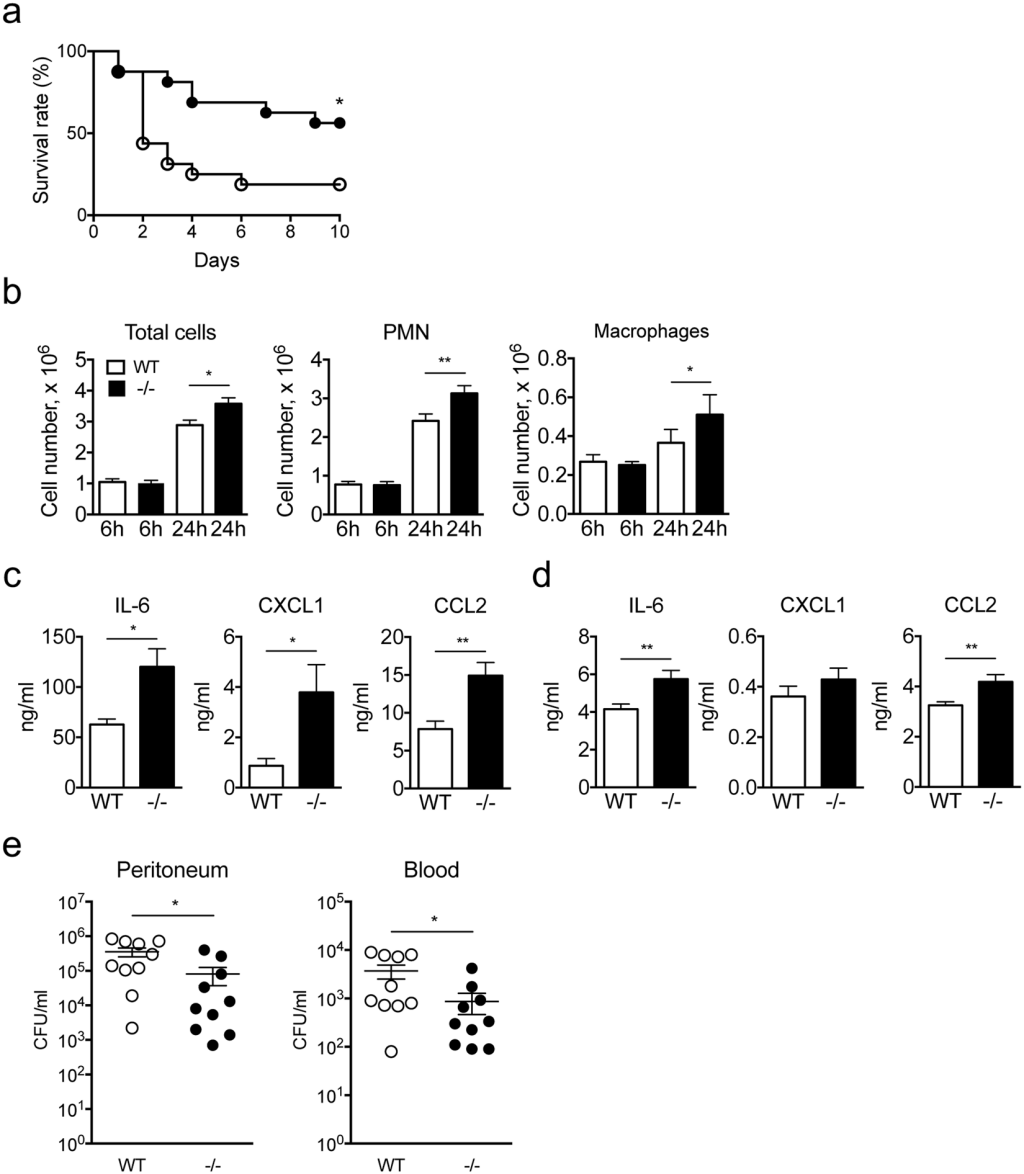
Improved survival and augmented leukocyte infiltration and bacterial clearance in Spred2^−/−^ mice after CLP. (**a**) The survival rates of WT and Spred2^−/−^ mice after CLP are shown. 18-G needles were used to puncture the cecum. Data is presented as the mean ± SEM. *p < 0.05. *n* = 16 per group. A summary of two individual experiments with similar results. (**b**) The number of neutrophils and macrophages in the peritoneal cavity was determined at 6 h or 24 h after CLP. (**c**) The levels of IL-6, CXCL1 and CCL2 in the peritoneal lavage fluids at 24 hours after CLP were measured by ELISA. (**d**) The levels of IL-6, CXCL1 and CCL2 in the serum at 24 hours after CLP were measured by ELISA. (**e**) The colony forming units (CFU) in 5 μl ascites or sera 24 h after CLP were examined. Ascites and sera were serially diluted and incubated in TSA plates containing 5% sheep blood for 24 h. Data represent the mean ± SEM. *n* = 10 per group. A summary of two individual experiments with similar results. *p < 0.05 and **p < 0.01.

To test the hypothesis that interfering Spred2 protects mice from PMS, we first induced septic peritonitis in WT and Spred2^−/−^ mice by CLP and examined the survival of mice. We used 18-G needles to puncture cecum. As shown in Fig. 1a, 9 out of 16 (56%) WT mice died by day 2 and 13 out of 16 (81%) died by day 10. In contrast, 7 out of 16 (44%) Spred2^−/−^ mice died. Thus, Spred2^−/−^ mice were protected from PMS caused by CLP.

Leukocytes, such as neutrophils and macrophages, are the first line of cells that encounter and respond to pathogens after CLP^7^. Therefore, we examined the infiltration of these leukocyte subsets into the peritoneum after CLP. Since the puncture with 18-G needles caused a heavy leak of intestinal contents that made the examination difficult, we used 22-G needles that induce milder PMS^19^. As shown in Fig. 1b, there was no significant difference in the numbers of total leukocytes, neutrophils or macrophages at 6 h after the surgery. At 24 h, however, the numbers of total leukocytes and each cell types infiltrating the peritoneum were higher in Spred2^−/−^ mice, compared to WT mice (1.3-fold increase for neutrophils and 1.4-fold increase for macrophages). The differences were modest but statistically significant.

We next measured the concentration of proinflammatory cytokines, including TNFα, IL-1β and IL-6, and chemokines, including CXCL1 and CCL2, in the peritoneal lavage fluid obtained at 6 or 24 h after CLP. There was no significant difference in the level of TNFα or IL-1β, in the peritoneal fluids between the two groups at both time points (data not shown). There was also no difference in the level of IL-6, CXCL1 and CCL2 at 6 h between the two groups (data not shown). However, the levels of IL-6, CXCL1 and CCL2 at 24 h were moderately but significantly elevated in the peritoneal fluids of Spred2^−/−^ mice relative to those of WT mice (Fig. 1c). The levels of these molecules in the serum at 24 h were much lower than those in the lavage fluids, but the levels of IL-6 and CCL2 were significantly elevated (Fig. 1d).

We also examined the bacterial load in the peritoneum and peripheral blood of mice after CLP. As shown in Fig. 1e, bacterial loads detected at 24 h after CLP in the peritoneum (left panel) and peripheral blood (right panel) of Spred2^−/−^ mice were significantly lower than those of WT mice, indicating that Spred2^−/−^ mice had a greater capacity to clear bacteria post CLP. Taken together, our results suggest that Spred2^−/−^ mice were protected from CLP-induced sepsis likely due to increased inflammatory responses against bacteria.

### Augmented chemokine production by Spred2^−/−^ macrophages

**Figure 2:**
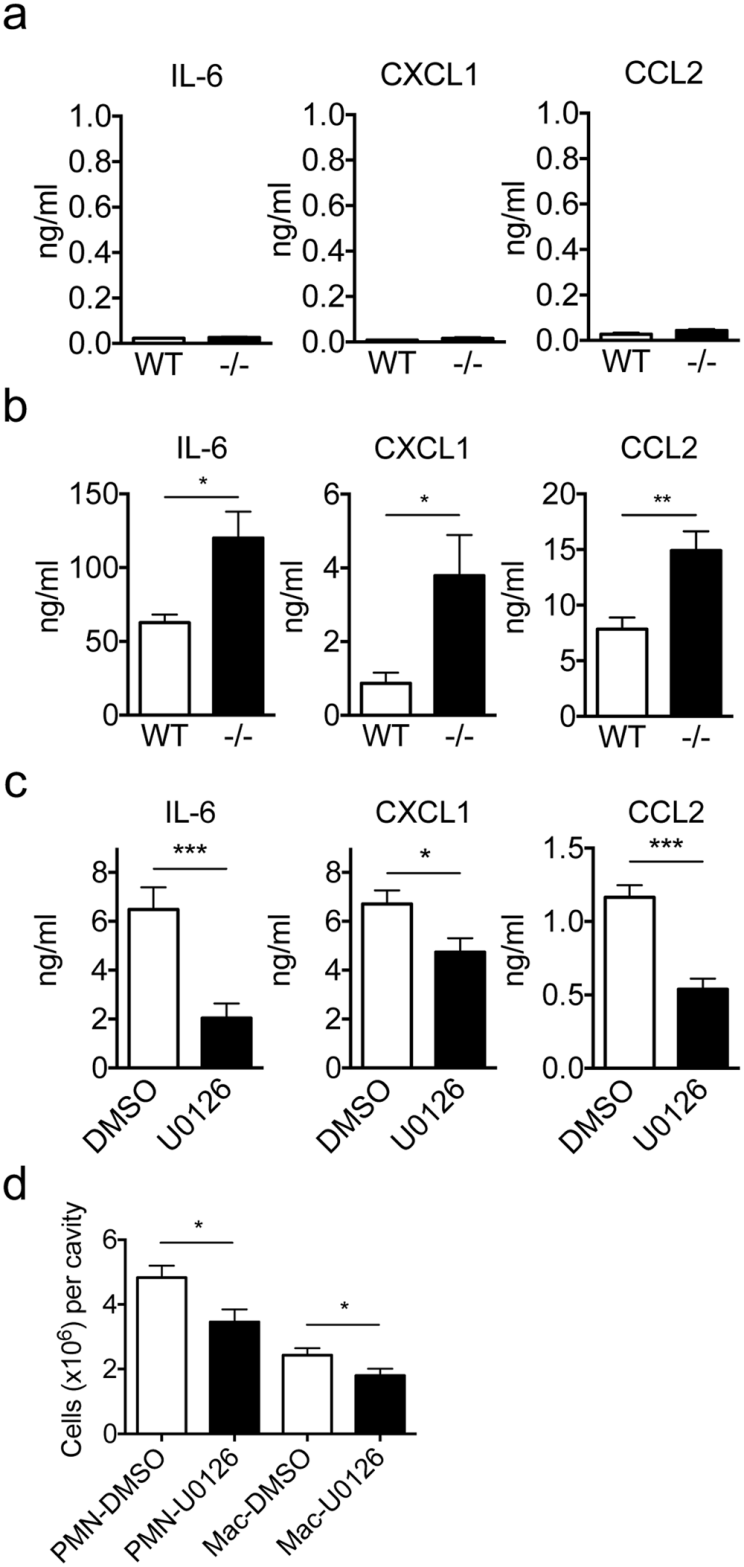
Increased production of IL-6, CXCL1 and CCL2 by LPS-activated peritoneal macrophages. The concentrations of IL-6, CXCL1 and CCL2 in the culture supernatants of peritoneal resident macrophages without activation (**a**) or activated by 100 ng/ml LPS for 24 h (**b**) were measured by ELISA. (**c**) Peritoneal resident macrophages from Spred2^−/−^ mice were activated by 100 ng/ml LPS for 24 h in the presence or absence of U0126 and the concentrations of IL-6, CXCL1 and CCL2 were measured by ELISA. (**d**) The number of infiltrating neutrophils (PMN) and macrophages (Mac) in the peritoneal cavity of Spred2^−/−^ mice, pretreated with DMSO or U0126, were counted at 24 h after CLP. Data represent the mean ± SEM. *n* = 4 for (a) and *n* = 10 for other experiments. *p < 0.05, **p < 0.01 and ***p < 0.001.

TLR4 plays a critical role in coordinating complex immune responses to bacterial infections^20^. To examine whether Spred2-deficiency affects the production of cytokines and chemokines, we isolated resident peritoneal macrophages from WT and Spred2^−/−^ mice and stimulated them with the TLR4 ligand LPS. There was only a low level of IL-6, CXCL1 or CCL2 in the culture supernatant when the cells were not activated, and there was no difference in the production of these molecules between WT and Spred2^−/−^ macrophages (Fig. 2a). As shown in Fig. 2b, however, macrophages from Spred2^−/−^ mice produced significantly higher levels of IL-6, CXCL1 and CCL2 than those from WT mice.

To examine whether this enhanced chemokine production by Spred2^−/−^ macrophages was mediated through the activation of ERK, macrophages were stimulated with LPS *in vitro* in the presence of U0126, an inhibitor of MAP Kinase Kinase (MAPKK or MEK1/2). U0126 significantly reduced LPS-induced production of IL-6, CXCL1 and CCL2 by macrophages (Fig. 2c). When U0126 was injected into WT mice before CLP, it significantly decreased CLP-induced neutrophil and macrophage infiltration at 24 h post CLP (Fig. 2d). These results indicate that the production of IL-6, CXCL1 and CCL2 was up-regulated in resident peritoneal macrophages of Spred2^−/−^ mice via activation of the MAPK pathway and that increased neutrophil and macrophage infiltration detected after CLP was due to increased activation of the MAPK pathway.

### Augmented phagocytosis of bacteria by Spred2^−/−^ macrophages

**Figure 3:**
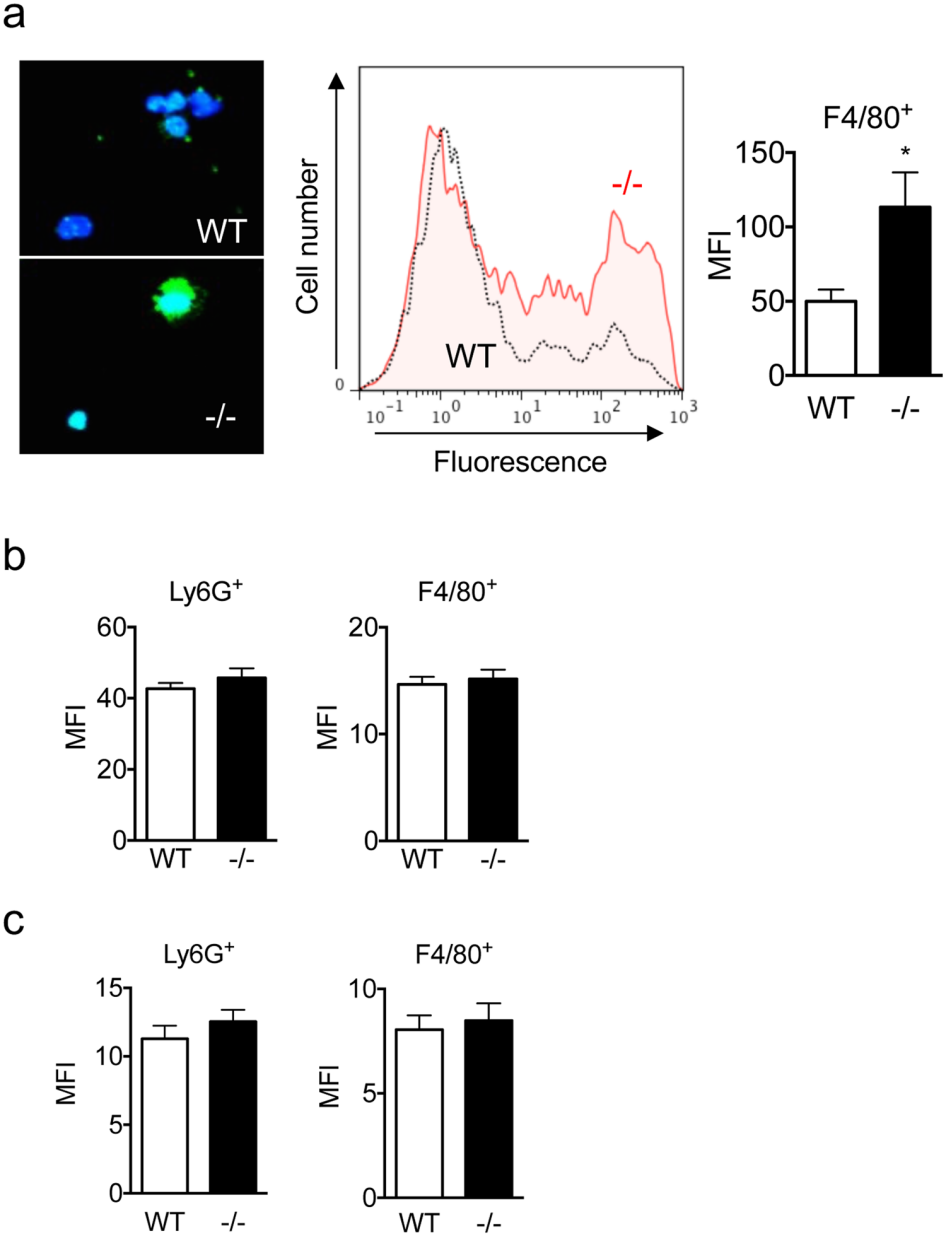
Augmented phagocytotic activity by Spred2^−/−^ peritoneal resident macrophages. (**a**) Peritoneal resident cells (1 × 10^6^/well) were incubated with AlexaFluor 488-conjugated *E.coli* bioparticles (1 × 10^6^/well) for 1 h and the level of phagocytosis was examined under fluorescence microscope (bioparticles are in green, and DAPI is in blue) and by flow cytometry. (**b**) TG-induced 16-h PEC (1 × 10^6^/well) were incubated with AlexaFluor 488-conjugated *E.coli* bioparticles (1 × 10^6^/well) for 1 h and gated by Ly6G or F4/80 staining, and the level of phagocytosis was evaluated by flow cytometry. Data represent the mean ± SEM, *n* = 12 per group. *p < 0.05. (**c**) TG-induced 16-h PEC or resident macrophages (1 × 10^6^/well) were incubated with 10 μL CellROX green reagent and ROS level in neutrophils or macrophages was evaluated by flow cytometry by gating with Ly6G- or F4/80-staining, respectively. Data represent the mean ± SEM (*n* = 5 mice/group). *p < 0.05.

It was previously demonstrated that infiltrating leukocytes clear bacteria from the peritoneum in this model^8,21^. A lower level of bacterial loads detected in Spred2^−/−^ mice can be ascribed to the increased number of infiltrating leukocytes, but it is also possible that Spred2^−/−^ leukocytes have a higher capacity to clear bacteria. To better understand the mechanism of increased bacterial clearance by Spred2^−/−^ mice, we examined the phagocytic activity of leukocytes. We first isolated peritoneal resident cells from untreated WT or Spred2^−/−^ mice and incubated them with Alexa Fluor 488-conjugated *Escherichia coli* bioparticles. As shown in Fig. 3a, peritoneal resident macrophages from Spred2^−/−^ mice exhibited augmented phagocytic activity relative to WT control as indicated by increased mean fluorescence intensity (MFI). The augmented phagocytic activity was ascribed to macrophages, as it was observed in Spred2^−/−^ F4/80^+^-cells, but not F4/80^−^-cells that were less then 5% of the total cells (data not shown).

We next examined the phagocytic activity of peritoneal exudate leukocytes. We isolated cells at 16 h after TG-injection (>85% neutrophils) and cells were incubated with Alexa Fluor 488-conjugated *Escherichia coli* bioparticles. Cells were gated with Ly6G or F4/80 and phagocytosis of bioparticules was analyzed by flow cytometer. Unlike resident macrophages, no difference was found between Ly6G^+^ or F4/80^+^ cells from WT and Spred2^−/−^ mice (Fig. 3b).

Phagocytosed bacteria are killed by leukocytes in association with the production of ROS^22^. Therefore, the production of ROS was examined using TG-elicited 16 h peritoneal exudate cells (PEC). As shown in Fig. 3c, there was no difference in the intensity of fluorescence between Ly6G^+^ WT and Spred2^−/−^ cells, and between F4/80^+^ WT and Spred2^−/−^ cell. Resident macrophages from WT and Spred2^−/−^ mice also produced similar amounts of ROS (data not shown). Thus, Spred2^−/−^ resident macrophages exhibited augmented phagocytic capacity but there was no difference in ROS production.

### Increased cell-surface expression of CR1/2 on Spred2^−/−^ macrophages via the ERK/MAPK pathway

**Figure 4:**
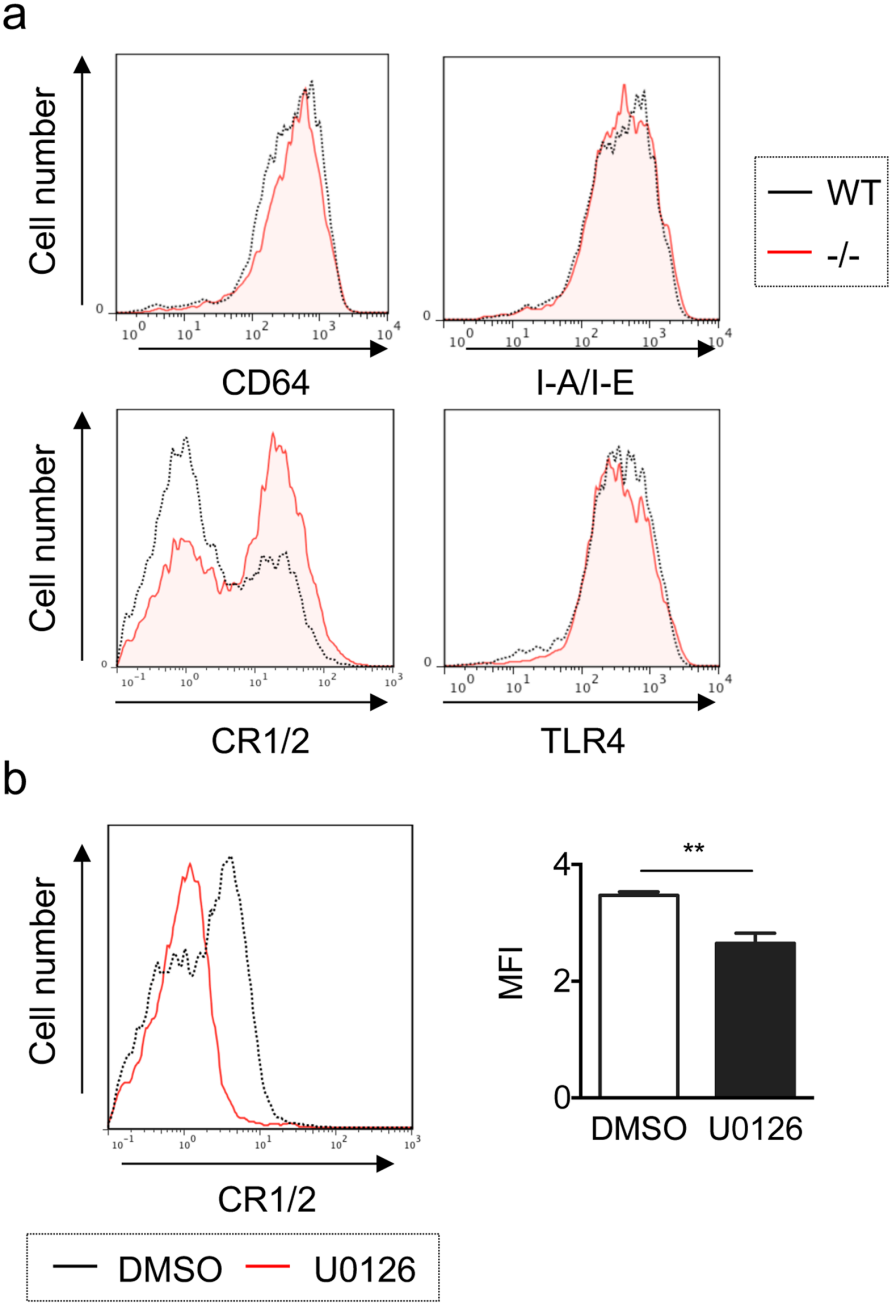
Augmented cell surface expression of CR1/2 by Spred2^−/−^ peritoneal resident macrophages. (**a**) Peritoneal resident cells (1 × 10^6^) were treated with 100 ng/ml LPS for 24 h and the cell-surface expression of CD64, I-A/I-E, CR1/2 and TLR4 was analyzed by flow cytometry. Cells were gated by the expression of F4/80. Representative results of three independent experiments are shown. (**b**) Peritoneal resident macrophages from Spred2^−/−^ mice (1 × 10^6^/well) were incubated with 100 ng/ml LPS in the presence or absence of U0126 (20 μl of 20 mM) for 24 h and the cell-surface expression of CR1/2 was analyzed by flow cytometry. Cells were gated on the expression of F4/80. Data represent the mean ± SEM. *n* = 5 per group. *p < 0.01.

Several cell-surface molecules, including CD64 (Fcγ receptor 1)^23^, I-A/I-E (MHC class ΙΙ)^24^, CR1/2^25^ and TLR4^26^, have been shown to play a role in the phagocytosis of bacteria. To understand the mechanisms of enhanced phagocytosis by Spred2^−/−^ resident macrophages, we evaluated the expression of these cell-surface molecules. The expression of these molecules was similar on unstimulated macrophages from either WT or Spred2^−/−^ mice (data not shown). However, upon stimulation with LPS, Spred2^−/−^ resident macrophages expressed significantly higher levels of CR1/2 than WT macrophages (Fig. 4a). The expression level of CR1/2 on freshly isolated resident macrophages was similar between the two strains (data not shown).

We next examined the involvement of the ERK/MAPK pathway in the regulation of CR1/2 expression by macrophages. Peritoneal resident macrophages from Spred2^−/−^ mice were stimulated with 10 ng/ml LPS in the presence or absence of U0126 for 24 hours and the expression of CR1/2 was analyzed by flow cytometry. Interestingly, U0126 significantly decreased the surface expression of CR1/2 on Spred2^−/−^ resident macrophages (Fig. 4b). Thus, the expression of CR1/2 was upregulated on Spred2^−/−^ resident peritoneal macrophages upon LPS stimulation via activation of the MAPK pathway.

### Increased inflammatory responses in Spred2^−/−^ mice via activation of CR1/2

**Figure 5:**
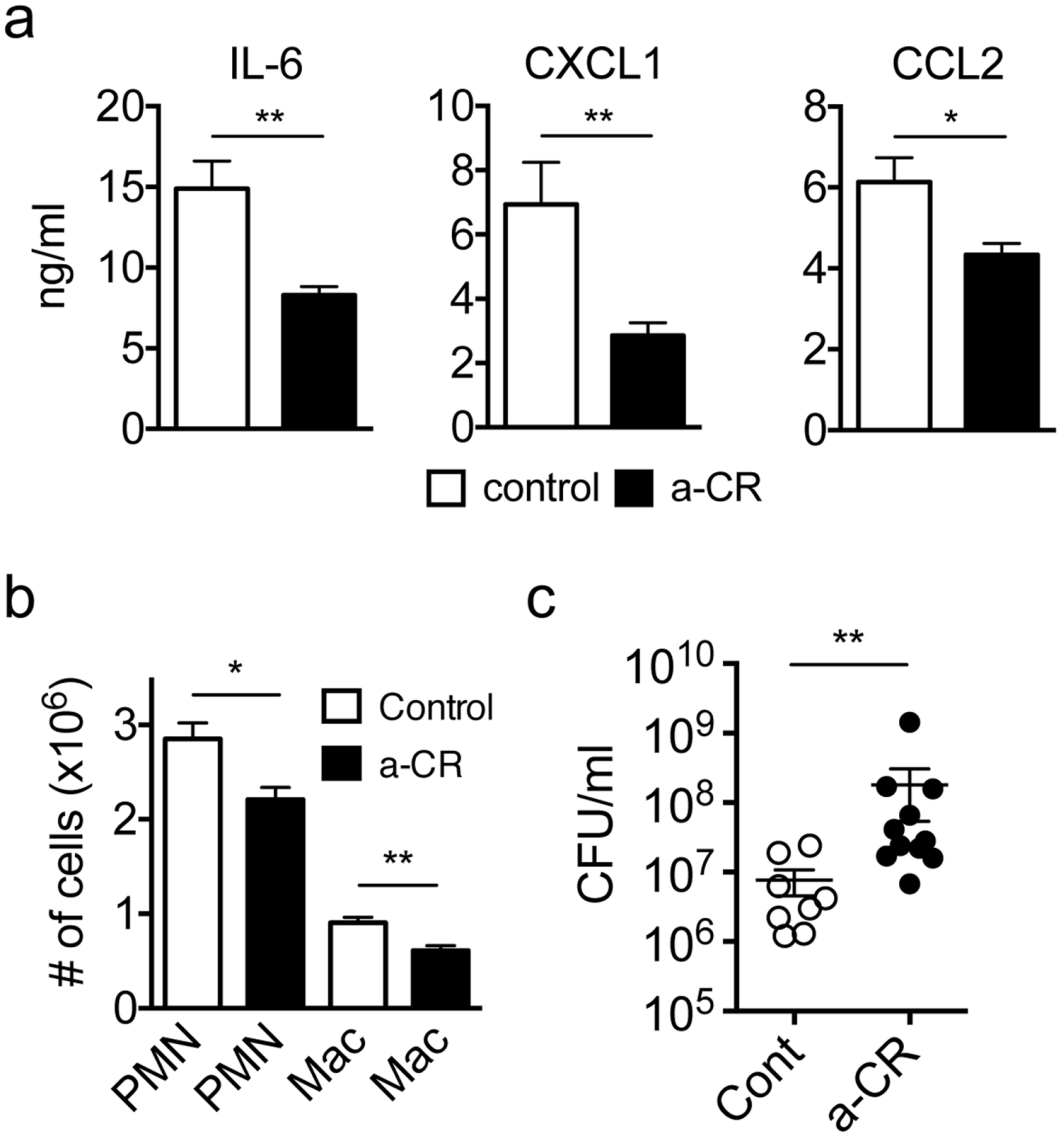
Reduced CLP-induced inflammation and bacterial clearance by the neutralization of CR1/2. (**a**) Spred2^−/−^ mice were intraperitoneally injected with anti-CR1/2 neutralizing antibody (3 μg/mouse) 2 h before CLP. The concentrations of IL-6, CXCL1 and CCL2 in the peritoneal fluids were measured by ELISA. Data represent the mean ± SEM. *n* = 10 per group. *p < 0.05 and *p < 0.01. (**b**) The number of neutrophil and macrophages infiltrating the peritoneal cavity was counted. Data represent the mean ± SEM. *n* = 8 for control, 11 for antibody-treated group. *p < 0.05 and **p < 0.01. (**c**) The bacterial loads in the peritoneal cavity were evaluated. Data represent the mean ± SEM. *n* = 8 for control, 11 for antibody-treated group. **p < 0.01.

To examine a potential role of CR1/2 in the augmented inflammatory responses detected in Spred2^−/−^ mice after CLP, a neutralizing anti-CR1/2 antibody (3 μg/mouse) was intraperitoneally injected into Spred2^−/−^ mice 2 h prior to CLP. As shown in Fig. 5a, injection of anti-CR1/2 antibody decreased the peritoneal levels of IL-6, CXCL1 and CCL2 (Fig. 5a) and peritoneal leukocyte infiltration after CLP (Fig. 5b). Furthermore, the bacterial burden was conversely increased (Fig. 5c). These findings strongly support the notion that the increased expression of CR1/2 on Spred2^−/−^ macrophages was responsible for the enhanced innate immune responses detected in Spred2^−/−^ mice in response to CLP.

## Discussion

We previously reported that Spred2-deficiency promoted the development of LPS-induced lung inflammation by up-regulating the production of proinflammatory cytokines and chemokines^18^. We also demonstrated that Spred2-deficiency exacerbated acetaminophen-induced hepatotoxicity via increased production of cytokines and chemokines by Kupffer cells and NK cells^27^. Since cytokine and chemokine responses are essential in the development of innate immunity against bacterial infections, including septic peritonitis, we hypothesized that Spred2-deficiency might protect mice from septic peritonitis. In the present study, we tested the hypothesis and found that Spred2^−/−^ mice, compared to WT mice, were indeed protected from PMS after CLP. The increased protection against PMS appeared to be due to the increased infiltration of inflammatory cells, such as neutrophils and macrophages, and also increased phagocytic activity by peritoneal resident macrophages. Increased cell-surface expression of CR1/2, which was regulated by the MAPK pathway, was at least partially responsible for the enhanced phagocytic activity. Although there have been numerous reports describing the roles of the MAPK pathway in innate and adaptive immune responses, this is the first report demonstrating the relationship between the MAPK pathway and cell-surface expression of CR1/2 by macrophages.

We detected elevated levels of cytokines and chemokines, including IL-6, CXCL1 and CCL2, in the peritoneal fluid of Spred2^−/−^ mice. IL-6 can be produced and released by T cells and macrophages and is considered to be a clinical marker of severe sepsis that could lead to high morbidity and mortality^28^. Our results, however, were incompatible with previous results because Spred2^−/−^ mice showed better survival in spite of a higher level of IL-6 production. This apparent discrepancy could be explained by the production of other cytokines/chemokines or the capacity of bacterial clearance. For example, CCL2 was previously shown to protect mice from endotoxemia. The administration of a neutralizing monoclonal antibody against CCL2 or genetic disruption of the CCL2 gene was associated with increased mortality rates in mice with sepsis^29,30^. Small differences between the level of these molecules produced by WT or Spred2^−/−^ macrophages were likely due to the puncture by 22-G needles which caused much milder PMS than that by 18-G needles.

Murine CR2 (CD21) and CR1 (CD35) are alternative splicing products of the *Cr2* gene. CR2 is expressed on B cells and follicular dendritic cells, whereas CR1 is expressed on various kinds of cells, including macrophages, erythrocytes, B and T lymphocytes^31^. CR1 and 2 bind to C3b and C3d, respectively, and were shown to play a pivotal role in innate and adaptive immunity, involving phagocytosis, complement regulation and maintenance of germinal center B cells and memory B cells^32,33^. Moreover, Donius *et al*. have reported that CR1/2-deficiency resulted in a higher mortality rate in a *Streptococcus pneumoniae*-induced pneumonia model^34^. These findings are consistent with our result that the expression of CR1/2 was enhanced in Spred2^−/−^ macrophages *in vitro* and responsible for increased inflammatory responses and bacterial clearance detected in Spred2^−/−^ *in vivo*.

The MAPK pathways are one of the most important signal transduction systems, which induce innate and adaptive immune responses. Inhibition or deletion of MAPKs, including ERK1/2, p38 and JNK, resulted in various immunological dysfunctions^11^. As described above, Spred2 is shown to inhibit the Ras/Raf/MAPK pathway by binding to Raf^27^. Raf is an important signaling molecule downstream of various growth factor receptors, but not of cytokine receptors. In this study, an increase in cytokine/chemokine production and cell-surface CR1/2 expression by Spred2^−/−^ macrophages in response to LPS was blocked by U0126, suggesting that Spred2 may be involved in the regulation of the MAPK pathway downstream of TLR4. The MAPK pathway is demonstrated to regulate the expression of proinflammatory cytokines/chemokines^11^ and also the trafficking of receptors, such as bradykinin B2 receptor^35^. However, the exact mechanisms whereby Spred2 plays a role in TLR4 signaling pathway remains unclear. Zhu *et al*. recently reported that tuberous sclerosis complex 1 (TSC1) inhibits M1 polarization of mouse macrophages by suppressing the Ras/Raf1/MEK/ERK pathway^36^. Further, TSC1^−/−^ macrophages produced higher levels of cytokines in response to LPS. Thus, Spred2 may control cytokine/chemokine production by inhibiting Raf. It is also possible that Spred2 suppresses TLR signaling by other unknown mechanism. Additional studies are necessary to determine exact mechanisms by which Spred2 controls the development of innate immune responses.

## Materials and Methods

### Reagents

Fluorescein isothiocyanate (FITC)- or phycoerythrin (PE)-conjugated anti-F4/80, purified or PE-conjugated anti-CD21/35 (CR 2/1, clone 7E9) and PE-conjugated anti-I-A/I-E were purchased from BioLegend (San Diego, CA). FITC-conjugated Ly6G (RB6-8C5), PE-conjugated anti-Toll-like receptor 4 (TLR4) and PE-conjugated anti-CD64 were from BD Biosciences (San Jose, CA). U0126 was purchased from Calbiochem (Merck KGaA, Darmstadt, Germany).

### Mice

Spred2^−/−^ mice on a C57BL/6J background were generated, as previously described^37,38^. No Spred2 expression was detected in peritoneal cells, spleens, lungs, livers and kidneys from Spred2^−/−^ mice, as assessed by TaqMan RT-qPCR (data not shown). C57BL/6J mice were used as the wild-type (WT) mice. These mice were bred at the Department of Animal Resources, Okayama University (Okayama, Japan). Male mice (10–12 weeks) were used in this study under specific pathogen-free conditions. The mice were housed in a temperature-controlled environment with a 12-h light/12-h dark cycle and allowed free access to water and food. All animal protocols were approved by the Animal Care and Use Committee of the Okayama University, and all experiments were performed in accordance with relevant guidelines and regulations.

### Induction of PMS

CLP surgery was performed as described in previous literatures^39,40^. In brief, mice were anesthetized by an intraperitoneal injection of ketamine (50 μg/kg body weight) and pentobarbital sodium (40 μg/kg body weight), and a 1 cm midline incision was made through the abdomen. The cecum was exposed and ligated with a 3-0 silk suture at half the distance between its distal pole and its base, and punctured through twice with an 18- or 22-gauge needle. The incision was closed with surgical clips. Mice were resuscitated with subcutaneous injection of 1 ml of warm sterile saline. Antibiotics were not given. The same surgical procedures were performed on sham animals without ligation and puncture. To evaluate the survival rate, mice were monitored for 10 days after CLP. In a different set of experiments, mice were anesthetized, bled, and euthanized 24 h after CLP. The peritoneal cavities were washed with 3 ml of cold sterile PBS, lavage fluids were harvested under sterile conditions. A 5-μl aliquot of lavage fluid and peripheral blood from each mouse were used for the assessment of bacteria load as described below. The remaining lavage fluids were centrifuged at 400 × *g* for 6 min at 4 °C. Cell-free peritoneal fluids were separated and stored at −80 °C until use. Cell pellets were resuspended in saline, and cell numbers were counted in a hemocytometer. Differential cell analyses were made by Diff-Quik staining of the smear slides or by flow cytometry.

### Determination of bacterial loads

Peritoneal lavage fluids or peripheral blood were serially diluted with sterile saline, and 5 μl of each diluted sample was plated on trypticase soy agar (TSA) plates with 5% sheep blood and incubated overnight at 37 °C, after which the number of aerobic bacterial colonies was counted. Data are presented as colony forming unit (CFU) ± SEM.

### Preparation of macrophages and neutrophils

Resident peritoneal cells were harvested from non-treated mice and 1 × 10^6^ cells were incubated for 1 h at 37 °C in antibiotics-free RPMI 1640 containing 5% FCS in 6-well culture plates. Each well was rinsed three times with sterile PBS, and adherent macrophages were cultured in RPMI-1640 supplemented with 5% FCS. Higher than 96% of adherent cells were F4/80-positive as determined by flow cytometer and used as macrophages. In a different set of experiments, mice were intraperitoneally injected with 1 ml of 4% thioglycollate (TG, Difco Laboratories, Detroit, MI) and PEC were harvested from the peritoneum 16 h after the injection. Neutrophils and macrophages were identified as Ly6G-positive and F4/80-positive by flow cytometer, respectively. Cell viability was routinely greater than 95%, as determined by trypan blue exclusion.

### Activation of macrophages

To evaluate the production of cytokines and chemokines by macrophages, adherent macrophages described above were stimulated with 100 ng/ml of LPS. After a 24 h-incubation, cell-free culture supernatants were harvested and stored at −80 °C until use. In a different set of experiments, peritoneal resident macrophages were incubated with 100 ng/ml LPS in the presence of 5 μM U0126 or vehicle control (DMSO) for 24 hours.

### Phagocytic activity

Phagocytic activity of peritoneal macrophages and neutrophils was determined using Alexa Fluor 488-conjugated *Escherichia coli* bioparticles (E-13231, Thermo Fisher Scientific K.K, Yokohama, Japan), as previously described^41,42^. Briefly, peritoneal macrophages or TG-induced 16 h PEC (1 × 10^6^ cells per well in a 6-well plate) were suspended in antibiotic-free RPMI 1640 with 5% FBS, and co-cultured with 1 × 10^6^ bioparticles for 1 h at 37 °C in a tissue culture plastic plate. Cells were then scraped off the surface and un-phagocytosed bioparticles that attached the cell surface were quenched by 0.4% trypan blue for 1 min. The fluorescent intensity of cells was measured by flow cytometry.

### Measurement of reactive oxygen species (ROS) production

For the measurement of ROS production, peritoneal resident macrophages or TG-induced 16-h PEC were stimulated with 100 ng/mL of LPS for 4 hours and then stained with 10 μL CellROX green reagent (5 μM, Thermo Fisher Scientific) or vehicle control (DMSO) for 30 minutes at 37 °C. Cells were then washed with PBS and analyzed on a flow cytometer. Neutrophils and macrophages were gated with Ly6G- and F4/80-staining, respectively.

### Flow cytometry

Cells were re-suspended in PBS supplemented with 2% FCS and 0.1% sodium azide (1 × 10^6^cells/100 μl), incubated with anti-CD16/32 antibody (BD biosciences, 5 min, 4 °C) to block Fc receptors, and stained with FITC- or PE-labeled antibodies. The intensity of fluorescence was measured on a MAQSQuant analyzer (Miltenyi Biotec, Germany). Compensation was performed in all experiments.

### Measurement of cytokine concentrations

The concentration of cytokines and chemokines in peritoneal lavage fluids or culture supernatants were measured by a standard method of sandwich ELISA, as previously described^6,43^. Captured antibodies, detection antibodies and recombinant cytokines were purchased from R&D Systems (Minneapolis, MN). ELISAs employed in this study did not cross-react with other murine cytokines available.

### Statistical analysis

All experiments were performed at least twice. Data were analyzed by the GraphPad Prism software (GraphPad Software, San Diego, CA, USA). Student *t* test or Kaplan-Meier survival method was utilized to determine the statistical significance. A value of *p* < 0.05 was considered statistically significant for all experiments. All values are presented as the mean ± SEM.

## References

[1] Angus DC, van der Poll T (2013). Severe sepsis and septic shock. N Engl J Med.

[2] Angus DC (2001). Epidemiology of severe sepsis in the United States: analysis of incidence, outcome, and associated costs of care. Crit Care Med.

[3] Rittirsch D, Flierl MA, Ward PA (2008). Harmful molecular mechanisms in sepsis. Nat Rev Immunol.

[4] Deutschman CS, Tracey KJ (2014). Sepsis: current dogma and new perspectives. Immunity.

[5] Matsukawa A, Kaplan MH, Hogaboam CM, Lukacs NW, Kunkel SL (2001). Pivotal role of signal transducer and activator of transcription (Stat)4 and Stat6 in the innate immune response during sepsis. J Exp Med.

[6] Matsukawa A (2003). Aberrant inflammation and lethality to septic peritonitis in mice lacking STAT3 in macrophages and neutrophils. J Immunol.

[7] Matsukawa A (2007). STAT proteins in innate immunity during sepsis: lessons from gene knockout mice. Acta Med Okayama.

[8] Watanabe H (2006). Overexpression of suppressor of cytokine signaling-5 in T cells augments innate immunity during septic peritonitis. J Immunol.

[9] Jarrar D (2002). Alveolar macrophage activation after trauma-hemorrhage and sepsis is dependent on NF-kappaB and MAPK/ERK mechanisms. Am J Physiol Lung Cell Mol Physiol.

[10] Kyriakis JM, Avruch J (2012). Mammalian MAPK signal transduction pathways activated by stress and inflammation: a 10-year update. Physiol Rev.

[11] Arthur JS, Ley SC (2013). Mitogen-activated protein kinases in innate immunity. Nat Rev Immunol.

[12] Zhang H, Moochhala SM, Bhatia M (2008). Endogenous hydrogen sulfide regulates inflammatory response by activating the ERK pathway in polymicrobial sepsis. J Immunol.

[13] Dumitru CD (2000). TNF-alpha induction by LPS is regulated posttranscriptionally via a Tpl2/ERK-dependent pathway. Cell.

[14] Riedemann NC, Guo RF, Ward PA (2003). The enigma of sepsis. J Clin Invest.

[15] Wakioka T (2001). Spred is a Sprouty-related suppressor of Ras signalling. Nature.

[16] Engelhardt CM (2004). Expression and subcellular localization of Spred proteins in mouse and human tissues. Histochem Cell Biol.

[17] Kato R (2003). Molecular cloning of mammalian Spred-3 which suppresses tyrosine kinase-mediated Erk activation. Biochem Biophys Res Commun.

[18] Xu Y (2014). Spred-2 deficiency exacerbates lipopolysaccharide-induced acute lung inflammation in mice. PLoS One.

[19] Ebong S (1999). Immunopathologic alterations in murine models of sepsis of increasing severity. Infect Immun.

[20] Deng M (2013). Lipopolysaccharide clearance, bacterial clearance, and systemic inflammatory responses are regulated by cell type-specific functions of TLR4 during sepsis. J. Immunol..

[21] Matsukawa A (2006). Absence of CC chemokine receptor 8 enhances innate immunity during septic peritonitis. FASEB J.

[22] Robinson JM (2008). Reactive oxygen species in phagocytic leukocytes. Histochem. Cell Biol..

[23] Danikas DD, Karakantza M, Theodorou GL, Sakellaropoulos GC, Gogos CA (2008). Prognostic value of phagocytic activity of neutrophils and monocytes in sepsis. Correlation to CD64 and CD14 antigen expression. Clin. Exp. Immunol..

[24] Nguyen HH, Tran BT, Muller W, Jack RS (2012). IL-10 acts as a developmental switch guiding monocyte differentiation to macrophages during a murine peritoneal infection. J. Immunol..

[25] Fällman M, Andersson R, Andersson T (1993). Signaling properties of CR3 (CD11b/CD18) and CR1 (CD35) in relation to phagocytosis of complement-opsonized particles. J. Immunol..

[26] Deng M (2013). Lipopolysaccharide clearance, bacterial clearance, and systemic inflammatory responses are regulated by cell type-specific functions of TLR4 during sepsis. J. Immunol..

[27] Wakabayashi H (2012). Spred-2 deficiency exacerbates acetaminophen-induced hepatotoxicity in mice. Clin Immunol.

[28] Mostafa Anower AK, Shim JA, Choi B, Sohn S (2012). Pretreatment with interleukin-6 small interfering RNA can improve the survival rate of polymicrobial cecal ligation and puncture mice by down regulating interleukin-6 production. Eur J Pharmacol.

[29] Gomes RN (2006). Increased susceptibility to septic and endotoxic shock in monocyte chemoattractant protein 1/cc chemokine ligand 2-deficient mice correlates with reduced interleukin 10 and enhanced macrophage migration inhibitory factor production. Shock.

[30] Matsukawa A (1999). Endogenous monocyte chemoattractant protein-1 (MCP-1) protects mice in a model of acute septic peritonitis: cross-talk between MCP-1 and leukotriene B4. J Immunol.

[31] Molnar E, Erdei A, Prechl J (2008). Novel roles for murine complement receptors type 1 and 2 I. Regulation of B cell survival and proliferation by CR1/2. Immunol Lett.

[32] Donius LR, Weis JJ, Weis JH (2014). Murine complement receptor 1 is required for germinal center B cell maintenance but not initiation. Immunobiology.

[33] Jacquet M (2013). Deciphering complement receptor type 1 interactions with recognition proteins of the lectin complement pathway. J Immunol.

[34] Donius LR, Handy JM, Weis JJ, Weis JH (2013). Optimal germinal center B cell activation and T-dependent antibody responses require expression of the mouse complement receptor Cr1. J Immunol.

[35] Khoury E, Nikolajev L, Simaan M, Namkung Y, Laporte SA (2014). Differential regulation of endosomal GPCR/β-arrestin complexes and trafficking by MAPK. J. Biol. Chem..

[36] Zhu L (2014). TSC1 controls macrophage polarization to prevent inflammatory disease. Nat. Commun..

[37] Nobuhisa I (2004). Spred-2 suppresses aorta-gonad-mesonephros hematopoiesis by inhibiting MAP kinase activation. J Exp Med.

[38] Taniguchi K (2007). Spreds are essential for embryonic lymphangiogenesis by regulating vascular endothelial growth factor receptor 3 signaling. Mol Cell Biol.

[39] Hubbard WJ (2005). Cecal ligation and puncture. Shock.

[40] Matsukawa A (2000). Expression and contribution of endogenous IL-13 in an experimental model of sepsis. J Immunol.

[41] Lehmann AK, Sornes S, Halstensen A (2000). Phagocytosis: measurement by flow cytometry. J. Immunol. Mehods.

[42] Kurotaki D (2011). CSF-1-dependent red pulp macrophages regulate CD4 T cell responses. J. Immunol..

[43] Matsukawa A (2005). Stat3 in resident macrophages as a repressor protein of inflammatory response. J Immunol.

